# Pseudomyxoma Peritonei Originating from an Intestinal Duplication

**DOI:** 10.1155/2013/608016

**Published:** 2013-08-19

**Authors:** Julie Lemahieu, André D'Hoore, Stijn Deloose, Raf Sciot, Philippe Moerman

**Affiliations:** ^1^Department of Imaging and Pathology, University Hospitals Leuven, Minderbroedersstraat 12, 3000 Leuven, Belgium; ^2^Department of Surgery, University Hospitals Leuven, Belgium; ^3^Department of Pathology, Jan Yperman Ziekenhuis, Ieper, Belgium

## Abstract

Alimentary tract duplications are rare congenital anomalies. They most often become symptomatic in childhood and rarely undergo malignant transformation. Pseudomyxoma peritonei (PMP) is an equally uncommon condition, most frequently originating from a primary appendiceal mucinous neoplasm. We report an extremely unusual case of PMP arising from an intestinal duplication. A 67-year-old woman presented with vague upper abdominal pain, and, unexpectedly, explorative laparoscopy revealed diffuse jelly-like peritoneal implants. The histopathological diagnosis of a low-grade PMP or “disseminated peritoneal adenomucinosis” was made. At that moment, no primary tumor was found. During later surgery, a cystic lesion located in the mesentery of the small bowel could be resected. Histologically, the cyst wall clearly showed the concentric layering of a normal bowel wall. The mucosa, however, displayed a diffuse low-grade villous adenoma. We concluded that this histological picture was most consistent with a small intestinal duplication, containing a low-grade villous adenoma. The adenoma caused a mucocele, which subsequently leaked or ruptured, giving rise to noninvasive mucinous peritoneal implants or low-grade PMP, also known as “disseminated peritoneal adenomucinosis” (DPAM).

## 1. Introduction

Alimentary tract duplications are rare congenital anomalies. They most often become symptomatic in childhood and rarely undergo malignant transformation. Pseudomyxoma peritonei (PMP) is an equally uncommon condition, most frequently originating from a primary appendiceal mucinous neoplasm. We report an extremely unusual case of PMP arising from an intestinal duplication.

## 2. Case Presentation

**Figure 1:**
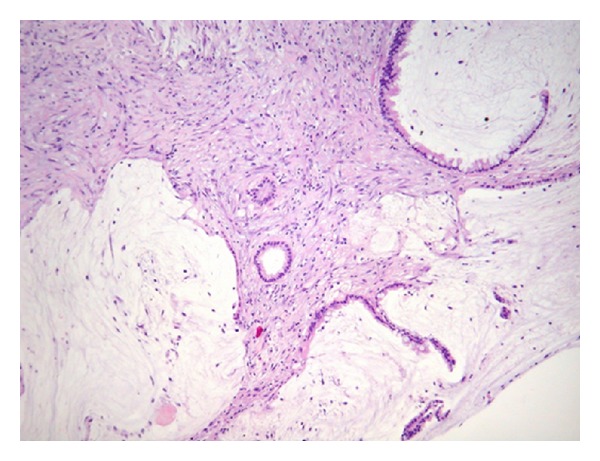
Noninvasive mucinous peritoneal implants or low-grade pseudomyxoma peritonei (PMP), also known as “disseminated peritoneal adenomucinosis” (DPAM).

Since 6 months, this 67-year-old woman complained of vague upper abdominal pain, without anorexia or weight loss. Her previous medical history was unremarkable, except for appendectomy in her adolescence, a caesarean section, and arterial hypertension. Physical examination, laboratory tests, and chest X-ray were normal. Esophagogastroscopy and ileocolonoscopy revealed no abnormalities. Abdominal CT showed a cystic lesion of 2 × 3 cm ventral to the aortic bifurcation, an omental nodule of 12 mm, and a small amount of fluid in the Douglas pouch. Supposedly, the cystic lesion was a necrotic adenopathy. Surprisingly, explorative laparoscopy revealed diffuse jelly-like peritoneal implants on the diaphragm, liver surface, and Douglas pouch. The small intestine and colon were free of implants. Uterus and ovaries had a normal appearance. Biopsies of the peritoneal implants showed dissecting pools of mucin, partially lined by a low-grade malignant intestinal type of epithelium (Figure 1). Immunohistochemically, the epithelial cells were strongly positive for CK20 and CDX2. Only rare cells stained weakly positive for CK7. The diagnosis was made of a low-grade PMP or “disseminated peritoneal adenomucinosis.” Despite further investigations, a primary tumor was not found. The patient was then referred to our tertiary center for cytoreductive surgery and hyperthermic intraperitoneal chemotherapy (HIPEC).

In our hospital, she underwent surgery with the purpose of debulking, but because of cardiac instability, the procedure had to be interrupted. However, a cystic lesion found in the mesentery of the small bowel could be resected.

**Figure 2:**
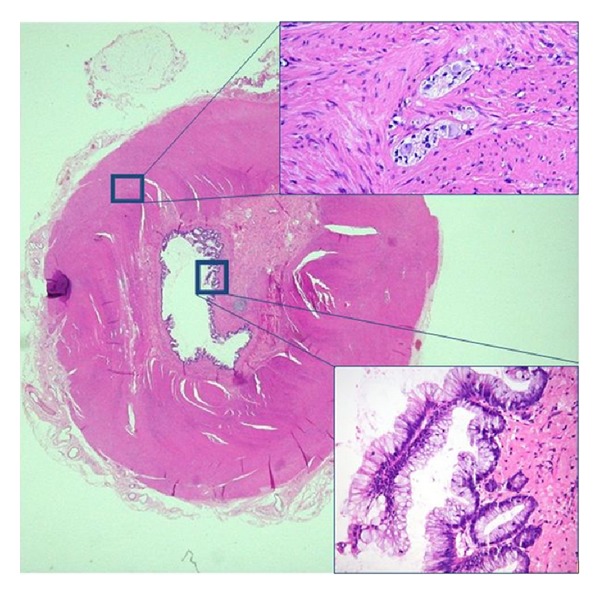
Intestinal duplication. The cyst wall clearly shows the concentric layering of a normal bowel wall. The tunica muscularis consists of two discrete smooth muscle layers with an intervening myenteric nerve plexus (upper inset). A submucosa and mucosa are also recognized. The mucosa displays a diffuse low-grade villous adenoma (lower inset).

On gross examination, the surgical specimen measured 3 × 2 × 2 cm, with a weight of 9 g. At one side, the surface was covered with a leaf of peritoneum. Sectioning revealed a thick-walled, mucin-filled cystic structure with a diameter of 1.5 cm. Histologically, there was extravasation of mucin in the surrounding connective tissue, indicating chronic leakage rather than acute rupture. The cyst wall clearly showed the concentric layering of a normal bowel wall (Figure 2). The tunica muscularis was thick and consisted of two discrete smooth muscle layers with an intervening myenteric nerve plexus. A submucosa and mucosa were also recognized. The mucosa, however, displayed a diffuse low-grade villous adenoma. We concluded that this histological picture was most consistent with a small intestinal duplication, containing a low-grade villous adenoma. The adenoma caused a mucocele, which subsequently leaked or ruptured, giving rise to noninvasive mucinous peritoneal implants or low-grade PMP, also known as “disseminated peritoneal adenomucinosis” (DPAM).

## 3. Discussion 

Alimentary tract duplication is a rare congenital malformation that involves the mesenteric side of the associated alimentary tract and shares a common blood supply with the native bowel. Several hypotheses have been formulated, but the etiology of this anomaly remains largely unknown. It usually becomes symptomatic in early childhood. Children with midgut duplication most often present with intussusception, acute appendicitis-like illness, small bowel obstruction, or gastrointestinal bleeding caused by the presence of ectopic gastric mucosa. In most patients, the diagnosis is not suspected before surgery [1]. On gross examination, alimentary tract duplications are spherical cysts or tubular structures, located in or next to the mesenteric side of a part of the gastrointestinal tract. Microscopically, they have a smooth muscle wall and are lined by gastrointestinal mucosa [2].

Intestinal duplications are rarely detected in adulthood and malignant transformation is an extremely rare complication, only diagnosed at advanced stage [3]. Reports of all cases with malignancy have been listed by Hata et al. [2] and Blank et al. [3]. The most frequent tumor type is adenocarcinoma, followed by squamous cell carcinoma and carcinoid tumor [2]. To our knowledge, only one case of PMP arising from an alimentary tract duplication has been reported in the English-language literature [4]. 

Clinically, PMP is a rare condition, difficult to diagnose because early symptoms are usually vague. The tumor cells produce abundant mucin that accumulates in the peritoneal cavity. Ensuing fibrosis of tissues and compression of vital organs gradually give rise to increasing abdominal complaints. Intraoperative findings include gelatinous intraperitoneal fluid collections and mucinous implants on the peritoneal surfaces and omentum. The sparing of the intestinal serosa, especially of the small bowel, is a classic observation which was also present in our case. In most cases, PMP originates from an appendiceal mucinous neoplasm. This theory is strongly supported by immunohistochemical staining with anticytokeratin antibodies. Most cases of PMP show strong positivity for CK20 and negative or only weak staining for CK7. This profile indicates a primary colorectal or appendiceal origin and militates against a primary ovarian tumor which is usually CK20 negative and CK7 positive. CDX2 is a fairly specific marker for the gastrointestinal origin of an adenocarcinoma. The primary tumor produces mucin, resulting in an appendiceal mucocele in case of obstruction of the appendiceal base. Rising intraluminal pressure will consequently lead to perforation and release of mucin and mucin-secreting tumor cells in the peritoneal cavity. Our patient underwent appendectomy in her adolescence. The pathology of the appendix is unknown, but considering the large time interval, any relation with the present pathology can be excluded. Although the primary appendiceal origin for most cases of PMP is now widely accepted, other exceptional origins (colon, stomach, pancreas, ovary, or urachus) have been convincingly reported, and intestinal duplication should be added to this short list.
